# From dynamic condensates to pathological assemblies: the phase behavior of tau and *α*-synuclein

**DOI:** 10.3389/fnmol.2026.1965177

**Published:** 2026-09-25

**Authors:** Suelen Camargo, Kevin Novelo, Scott D. Ryan

**Affiliations:** 1 Department of Clinical Neuroscience, University of Calgary, Calgary, AB, Canada; 2 Department of Physiology and Pharmacology, University of Calgary, Calgary, AB, Canada

**Keywords:** amyloid aggregation, biomolecular condensates, LLPS, amyloid generation, synuclein alpha, tau

## Abstract

Neurons rely on precise protein folding, trafficking, and turnover to maintain synaptic function. Disruption of proteostasis promotes misfolded proteins that impair axonal transport, mitochondrial function, and signalling, contributing to neuronal loss. Although neurodegeneration has traditionally focused on stable amyloid deposits, many disease-associated proteins contain intrinsically disordered regions (IDRs) that can organize through liquid–liquid phase separation (LLPS). LLPS concentrates proteins and nucleic acids into dynamic condensates that regulate cellular processes, but these adaptive interactions can also promote condensate aging and less reversible, aggregation-prone states. Here, we review how sequence-encoded properties of tau and α-synuclein (α-syn) link physiological condensation to pathological protein assembly, while emphasizing that LLPS and amyloid formation are related but not obligatorily linked.

## Introduction

Liquid–liquid phase separation (LLPS) occurs when macromolecules demix from a homogeneous solution to form a biomolecule-rich dense phase that coexists with a dilute phase. This transition is favoured when macromolecules condense into a biomolecule-rich phase that can appear as liquid-like droplets that coexist with a surrounding dilute phase. Phase separation occurs when the concentration and interaction valency of proteins or nucleic acids exceed condition-dependent thresholds set by temperature, pH, ionic strength, crowding, and molecular partners (Alberti et al., 2019). The resulting condensates provide cells with a dynamic means of organizing biochemical reactions without enclosing them within a membrane (Shin and Brangwynne, 2017).

LLPS-induced condensates are membraneless assemblies formed through the reversible condensation of proteins, RNAs, and other biomolecules into dense, yet dynamic, molecular environments via non-stoichiometric interactions (Mukhopadhyay, 2020). Their compositions are selective rather than indiscriminate, and many components exchange rapidly with the surrounding dilute phase (Banani et al., 2017; Alberti et al., 2025). Condensate architecture is stabilized by weak, transient, and multivalent interactions involving intrinsically disordered regions (IDRs), which provide the flexibility required for dynamic molecular interactions (Garaizar et al., 2020). Condensation therefore reflects a cooperative network of scaffold–scaffold, scaffold–client, and client–client interactions rather than a property of disorder alone (Choi et al., 2025). Moreover, condensates can occupy a continuum of material states, ranging from highly fluid, liquid-like droplets to gel-like or solid-phase assemblies (Mitrea et al., 2022). Condensate maturation is not synonymous with amyloid formation and can instead produce reversible condensates, gel-like or non-amyloid assemblies, altered conformational states, or amyloid fibrils. The material state depends on the strength, valency, and lifetime of the underlying molecular interactions, which can be modulated by factors such as ATP concentration, pH, temperature, mutations, and post-translational modifications (PTMs) (Li et al., 2026). This sensitivity enables condensates to rapidly adapt their physical properties in response to changes in the cellular environment while maintaining their biological functions (Wang et al., 2024b). Furthermore, recent imaging technologies have enabled real-time monitoring of condensate formation, dynamics, and maturation (Xue et al., 2025).

Under physiological conditions, biomolecular condensates create specialized environments that enhance biochemical and enzymatic reactions, promote low-valency molecular interactions, and protect cellular components from stress (Wang et al., 2024a, 2024b). Furthermore, the unique physicochemical and mechanical properties of condensates enable them to generate forces that remodel or bend cellular membranes, influence cellular architecture, and act as selective molecular filters (Nott et al., 2015; Alberti et al., 2019; Mondal and Baumgart, 2023). This spatial organization enables cells to rapidly regulate biochemical processes by controlling molecular concentration, accessibility, and interactions (Su et al., 2021). Condensates can also buffer protein fluctuations by storing and releasing molecules while selectively recruiting protein and RNA to control their localization. As a result, cellular pathways remain highly responsive and adaptable to sudden changes in environmental conditions (Figure 1) (Alberti et al., 2019; Yang et al., 2023).

**Figure 1:**
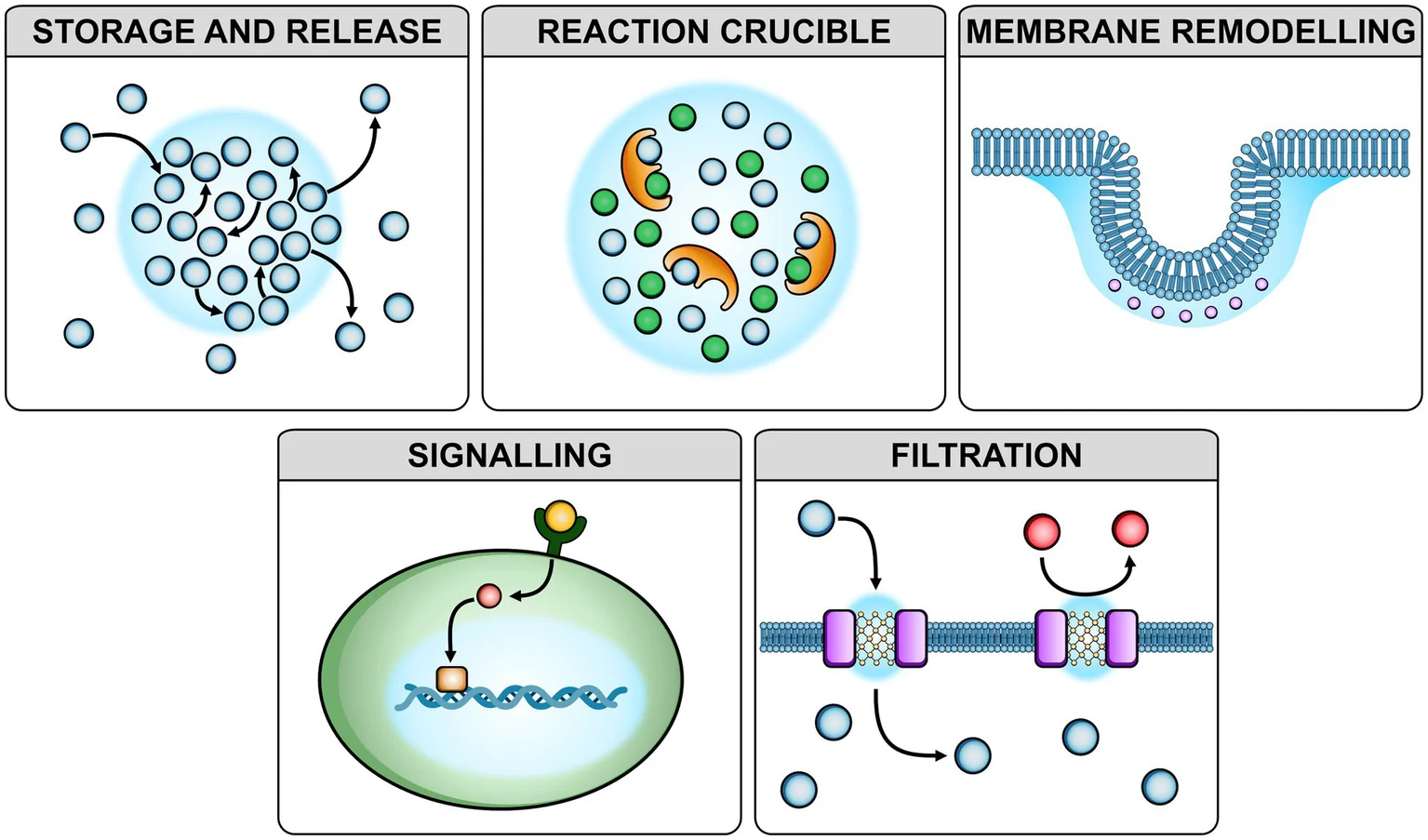
Biomolecular condensates organize cellular biochemistry by dynamically sensing and responding to environmental cues. They contribute to cellular homeostasis by reversibly storing and buffering proteins, concentrating or sequestering enzymes, substrates, and signaling molecules to regulate reaction rates and pathway activity, and selectively recruiting proteins and nucleic acids into membraneless compartments such as nucleoli, stress granules, and P-bodies. Beyond biochemical regulation, condensates can generate mechanical forces that influence membrane curvature and organelle architecture, while also establishing selective physicochemical environments that control molecular partitioning and exchange. Illustration created using Affinity Suite.

Despite their essential roles in cellular organization, condensates require tight regulation. Genetic mutations, aging, or environmental changes can alter their material properties, resulting in a drift in physical state that can transition from reversible LLPS to irreversible liquid–solid phase separation (LSPS) (Li et al., 2024). The solid-like condensates formed are often resistant to normal cellular clearance mechanisms, and their accumulation over time can seed protein aggregation, disrupting organelle function and facilitating fibril seeding (Figure 2) (Vendruscolo and Fuxreiter, 2022; Visser et al., 2024). Importantly, condensate maturation does not inevitably lead to amyloid formation; depending on protein sequence, interaction networks, and environmental conditions, condensates may remain reversible, transition into gel-like or other non-amyloid assemblies, undergo conformational rearrangements, or progress to amyloid fibrils (Lucas et al., 2026). Dysregulated condensation is therefore best viewed as one potential route to pathological protein assembly in Alzheimer’s disease (AD), Dementia with Lewy bodies and Parkinson’s Disease (PD) (Ambadipudi et al., 2017; Zbinden et al., 2020; Mukherjee et al., 2023).

**Figure 2:**
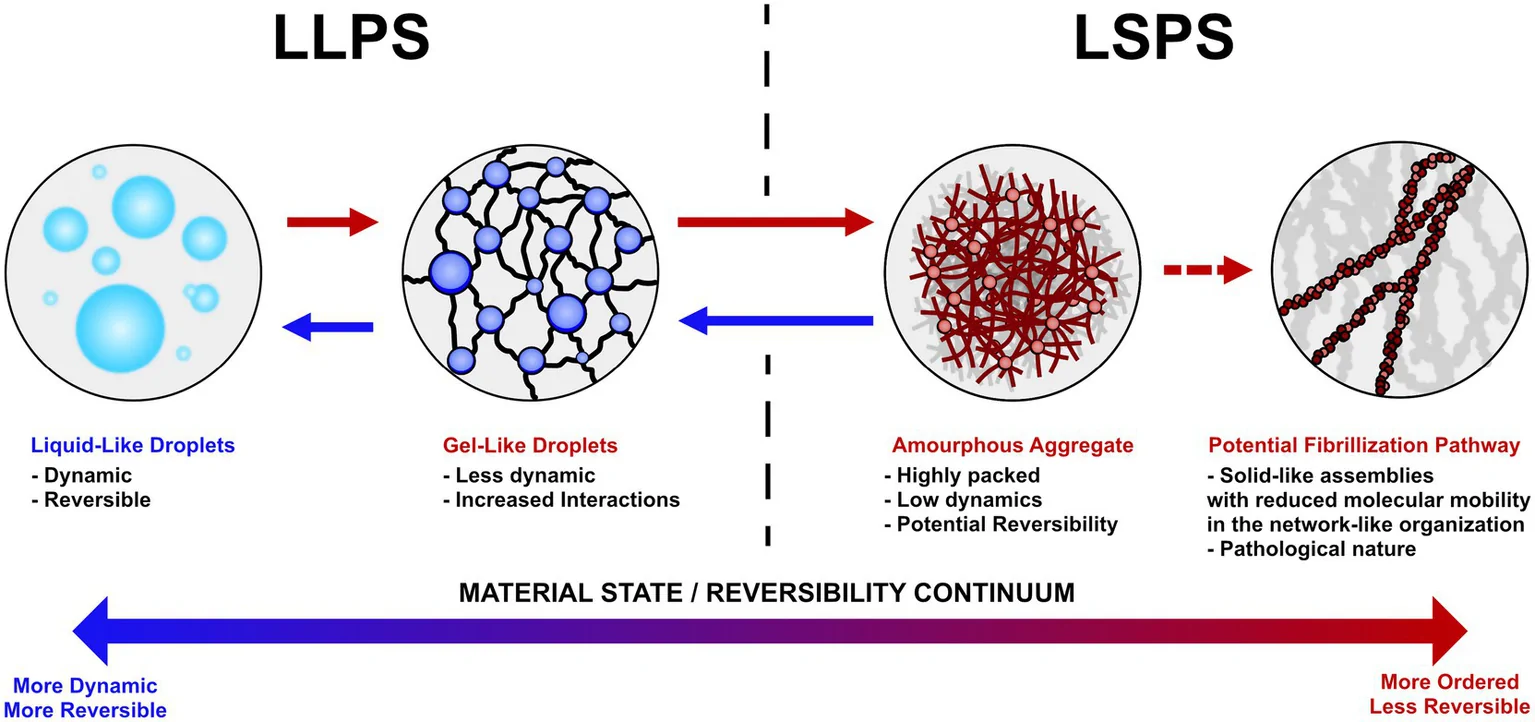
Liquid-like biomolecular condensates can mature into less dynamic, gel-like or solid-phase assemblies through changes in molecular interactions, condensate composition, and environmental conditions. Proteins may nucleate and form fibrils within condensates or at their interfaces, coupling condensate maturation to pathological aggregation. This maturation is often accompanied by reduced molecular mobility, increased structural organization, and altered material properties. However, not all condensates undergo fibrillation; some mature into reversible gel-like and liquid-like states, whereas others mature through pathways that are only partially dependent on fibril formation. Illustration created using Affinity Suite.

## Protein sequence features underlying LLPS behavior

Protein sequence and architecture play fundamental roles in governing the molecular interactions that underlie biomolecular condensation (Saar et al., 2021). Depending on their amino acid composition, proteins can adopt diverse structural conformations, ranging from highly ordered, folded domains to flexible IDRs (Trivedi and Nagarajaram, 2022) Intrinsically disordered proteins (IDPs) lack a single stable tertiary structure across all or most of their sequence, whereas IDRs are flexible segments embedded within otherwise structured proteins (Trivedi and Nagarajaram, 2022). In both cases, amino acid composition and sequence patterning shape conformational ensembles and molecular interactions (Trivedi and Nagarajaram, 2022). Phase-separation competence is therefore encoded not simply by disorder, but by the number, chemistry, and arrangement of interaction sites (Bianchi et al., 2020). Phase-separating IDRs are typically characterized by low-complexity sequences enriched in polar, charged, and aromatic residues that are arranged in biased compositions rather than well-defined folded motifs (Tripathi and Gupta, 2026). These sequence features promote phase separation by enabling multivalent interactions, including cation–π, π–π, electrostatic, and hydrophobic interactions. Together, these interactions promote condensate formation and regulate the assembly, segregation, and packing of biomolecules within biochemical compartments (Ahmad et al., 2022; Calvário et al., 2025; Li et al., 2025). Groups of residues that mediate cohesive interactions are known as “stickers,” while the intervening flexible regions, termed “spacers,” regulate the strength and dynamics of these interactions (Choi and Pappu, 2020). This “sticker–spacer” architecture allows proteins dispersed in a homogeneous aqueous solution to undergo demixing and form liquid-like condensates (Choi and Pappu, 2020; Ibrahim et al., 2023; Liu et al., 2025; Maristany et al., 2025; Zhou, 2025). Beyond governing the onset of phase separation, the number and arrangement of stickers also influence the spatial organization of different proteins within multicomponent condensates. In the sticker–spacer framework, increasing effective sticker valency strengthens network connectivity, generally lowering the saturation concentration and shifting the phase boundary so that condensates form at lower protein concentrations. Differences in protein valency drive the internal organization of biomolecular condensates, with high-valency proteins enriching the core and low-valency proteins accumulating at the interface. This thermodynamic partitioning provides a mechanistic basis for scaffold–client behavior and demonstrates how subtle changes in molecular interactions can regulate condensate architecture, multilayering, and phase behavior (Sanchez-Burgos et al., 2021).

Not all IDPs can undergo LLPS under physiological conditions, suggesting that specific molecular features determine phase separation propensity. Machine-learning analyses have identified key sequence features governing LLPS propensity, including hydrophobicity, aromatic content, charge distribution, charge patterning, and conformational expansion of IDRs (von Bülow et al., 2025). Molecular partners can be equally important as physiological LLPS relies on cofactors or scaffolds rather than intrinsic protein properties. For instance, tau is reported to undergo scaffold-mediated LLPS with RNA or polyanions and can also form homotypic condensates, whereas α-synuclein (α-syn) can undergo homotypic LLPS under appropriate solution conditions (Lin et al., 2019; Najafi et al., 2021; Zappone et al., 2025). Biomolecular condensates are essential for cellular function, but their dysregulation can contribute to neurodegenerative disease through aberrant phase transitions of RNA-binding proteins. RNA plays a key regulatory role in condensate assembly and dynamics, either promoting condensation through scaffolding effects or preventing pathological aggregation (Kiebler and Bauer, 2024). RNA therefore exerts a context-dependent effect. Distinct RNA species differentially regulate condensate formation and composition, with long, flexible RNAs acting as scaffolds that promote multivalent interactions among RNA-binding proteins (RBPs), driving phase separation at intermediate protein-to-RNA ratios. In contrast, short RNAs or high RNA concentrations tend to suppress condensate assembly by limiting RBP multimerization and favoring monomeric RNA–protein interactions (Asamitsu and Shioda, 2021; Mann and Donnelly, 2021).

Tau is a microtubule-associated protein composed of four distinct structural domains (Dangi et al., 2020; Zeng et al., 2021; Limorenko and Lashuel, 2022). Encoded by the microtubule-associated protein tau (MAPT) gene on chromosome 17q21.31, tau exhibits extensive isoform diversity through the alternative splicing of exons 2, 3, and 10. As a result, there are six major brain-associated tau isoforms, defined by the presence or absence of the N-terminal repeats (0 N, 1 N, or 2 N) and the second microtubule-binding repeat (3R or 4R), of which 2N4R tau is the longest at 441 amino acids (Buchholz and Zempel, 2024). The N-terminal projection domain (NTD) of the 2N4R tau isoform forms an acidic, amphipathic region with a negative net charge (Derisbourg et al., 2015; Barbier et al., 2019). Together with the positively charged proline-rich domain (PRD), this region supports long-range electrostatic interactions that regulate full-length tau441 conformation and LLPS (Boyko et al., 2019). The PRD contains tubulin-interaction sites and several disease-relevant phosphorylation sites, including T181, T217, T231, and S235 (Acosta et al., 2025). The PRD provides multivalent contacts that promote tau condensation and microtubule organization. However, studies in cells and with full-length 2N4R tau indicate that LLPS arises from a distributed network of interactions rather than being driven solely by the PRD (Zhang et al., 2020; Wen et al., 2025). Studies using tau fragmentation show the microtubule-binding domain (MTBD) comprises four imperfect repeats in 4R tau and contains the amyloidogenic PHF6* (275–280) and PHF6 (306–311) motifs (Ammar Khodja et al., 2024). In both full-length and disease-associated tau mutants, these motifs increase β-sheet propensity and can promote amyloid nucleation within condensates, including in the context of the ΔK280 mutation (Wegmann et al., 2018; Chen et al., 2025). The C-terminal tail remains disordered and modulates tau protein–protein interactions and aggregation (Kanmert et al., 2015; McKibben and Rhoades, 2019). The PRD and MTBD also cooperate in tubulin binding and polymerization (McKibben and Rhoades, 2019). Under physiological conditions, full-length tau condensates arise from cooperative electrostatic interactions in the unstructured N-terminal region and hydrophobic interactions within the C-terminal domain and MTBD, which promote condensate stabilization via β-sheet structures. Indeed, electrostatic charge is a critical determinant of tau LLPS, as electrostatic interactions within the unstructured NTD region drive phase separation and are sensitive to counterions. Hyperphosphorylation and pro-aggregation mutations that alter tau charge distribution and increase β-sheet propensity further promote both LLPS and the tendency to aggregate, coupling condensate formation to β-sheet-rich tau assemblies (Wegmann et al., 2018).

The relationship between protein sequence, intrinsic disorder, and LLPS is also exemplified by α-syn, an IDP that accumulates in Lewy bodies. α-Syn is a 140-amino-acid IDP comprising three commonly defined regions: an N-terminal membrane-binding domain (NTD; residues 1–60), the central non-amyloid-β component (NAC; residues 61–95), and an acidic C-terminal domain (CTD; residues 96–140) (Sharma and Burré, 2023). The N-terminus adopts an amphipathic α-helix upon binding anionic phospholipids. This conformational plasticity supports synaptic-vesicle association and contributes to SNARE-complex assembly and neurotransmitter release (Ranjan, 2026). This domain mediates synaptic vesicle association and is important for both physiological function and LLPS stabilization (Gilmozzi et al., 2020; Ray et al., 2020; Stephens et al., 2020). The hydrophobic NAC region is highly hydrophobic and aggregation-prone (Giasson et al., 2001), despite mediating membrane association and axonal localization of α-syn (Anderson et al., 2020). Moreover, the NAC and NTD regions have been described as playing roles in initiating condensate formation during LLPS (Ray et al., 2020; Sawner et al., 2021). The CTD is highly acidic and glutamate-rich, remaining intrinsically disordered even upon membrane binding. This domain binds metal ions, polycations, and nucleic acids (Kim et al., 2002; Fusco et al., 2014; Mahapatra and Newberry, 2024). While specific environmental factors (such as low pH and increased salt concentration) can accelerate LLPS, truncation of the C-terminus can weaken intramolecular interactions, promoting an elongated structure with greater NAC exposure. Therefore, the same molecular features that promote condensation can also accelerate the maturation of condensates into amyloid aggregates (Sternke-Hoffmann et al., 2024). Moreover, condensate interfaces may accelerate fibril formation by concentrating aggregation-prone proteins and generating charged microenvironments that favor amyloid nucleation (Hoffmann et al., 2023; Yamaguchi et al., 2025). Indeed, this observation is consistent between both tau and α-syn, suggesting that the same sequence features that enable LLPS in tau and α-syn may also drive pathological transitions.

## Bridging physiological and pathological phase behavior

The structural features that enable tau and α-syn to perform condensate-dependent functions also contribute to their susceptibility to pathological transitions. Tau commonly undergoes complex coacervation with RNA and other polyanions (Zhang et al., 2017; Lin et al., 2021; Abasi et al., 2024), whereas homotypic α-syn LLPS is often observed *in vitro* under crowding, elevated concentration, acidic pH, or altered ionic conditions (Sternke-Hoffmann et al., 2024; Wasim et al., 2024; Sarkar et al., 2025). This distinction arises from their structural differences: the PRD and MTBD of tau provide multivalent interaction platforms that promote LLPS by engaging in cooperative interactions with other proteins, nucleic acids, and chromatin-associated components, thereby modulating the phase behavior of these systems (Abasi et al., 2024) Tau LLPS is a context-dependent process in which the nature of condensate-driving interactions determines whether it supports physiological regulation or promotes pathological aggregation. Electrostatic interactions with RNA can maintain reversible, dynamic condensates, whereas hydrophobic interactions and condensate maturation can drive β-sheet formation and amyloid fibril formation. Thus, phosphorylation may regulate tau phase behavior by altering charge distribution and modulating these interactions (Zhang et al., 2017; Boyko et al., 2019; Lin et al., 2021).

By contrast, α-syn undergoes LLPS through sequence features that promote complementary interactions within its flexible and dynamic structure (Sun et al., 2025). The NTD of α-syn promotes LLPS through multivalent electrostatic and π-π interactions, which counterbalance the repulsive effects of its acidic CTD. The central NAC region promotes phase separation by providing hydrophobic interactions that favor α-syn self-association, enabling coacervation without external polyanion scaffolds (Ray et al., 2020; Sawner et al., 2021; Dada et al., 2023). α-syn condensate maturation into amyloid fibrils is sensitive to the properties of the condensate interface, where enhanced condensate–surface interactions promote faster maturation. Alterations in the N-terminal domain can modify surface wetting properties, accelerating the transition from liquid-like condensates to solid condensates consisting of amyloid fibrils (Thrush et al., 2025). These findings highlight the importance of condensate interfaces in regulating pathological aggregation. Conversely, β-Synuclein (β-Syn), an α-syn homolog, can modulate this behavior by co-condensing with α-syn and limiting condensate growth and maturation, suggesting a protective role in regulating α-syn phase transitions (Xu et al., 2025).

## Tau and α-SYN LLPS in disease

Evidence from recombinant systems and cellular models supports a temporal relationship between LLPS and aggregation for both tau and α-syn, wherein LLPS is reported to precede aggregation in both AD and PD model systems (Mukherjee et al., 2023). In tauopathy models, liquid tau condensates have been observed *in vitro* and in-cell before neurofibrillary tangles appear, suggesting condensate maturation is an early event in disease progression (Bancher et al., 1989; Kang et al., 2021; Boyko and Surewicz, 2022). Similarly, α-syn liquid condensates are proposed to form in presynaptic regions before fibril deposition, suggesting LLPS may initiate neurodegeneration rather than result from it (Hardenberg et al., 2021; Dada et al., 2023; Piroska et al., 2026). However, direct evidence that presynaptic condensates are obligatory precursors of Lewy bodies *in vivo* remains limited (Hardenberg et al., 2021; Dada et al., 2023; Piroska et al., 2026). Indeed, in the case of α-syn, Sternke-Hoffmann et al. (2024) have shown that LLPS and aggregation exhibit distinct salt dependencies, with elevated salt concentrations favouring fibril formation by lowering protein solubility and strengthening hydrophobic interactions, suggesting that condensates and fibrils can form independently. Under certain conditions, LLPS may even protect against aggregation by maintaining protein dynamics or promoting aggregate dissolution (Sternke-Hoffmann et al., 2024). Similarly, studies of tau LLPS suggest that condensate formation does not necessarily dictate amyloid aggregation. Tau complex coacervation with polyanions can occur independently of aggregation, as only specific polyanions or disease-associated mutations enhance amyloid formation (Lin et al., 2020).

Although tau pathology is traditionally associated with neurofibrillary tangles, the emerging concept of tau LLPS provides new insights into the role of condensates in regulating tau function and pathological aggregation (Islam et al., 2024). Studies using full-length tau (Abasi et al., 2024) and tau fragments (Powell et al., 2024), derived from the 2N4R sequence, show that as fibrils form within condensates, they recruit monomers, creating self-propagating aggregates that accumulate as neurofibrillary tangles. These tangles sequester soluble tau, disrupting microtubule stability, impairing axonal transport, and ultimately causing neuronal death (Abasi et al., 2024; Islam et al., 2024; Powell et al., 2024). Hochmair et al. (2022) showed that molecular crowding and RNA act synergistically to support the condensation of recombinant human Tau441 under physiological buffer conditions. These condensates developed seeding activity over time even when their material properties and packing differed, indicating that pathological conformers are not dependent on a simple liquid-to-solid trajectory (Hochmair et al., 2022). Using the 0N4R tau isoform, Ash et al. (2021) showed that RNA and the RNA-binding protein TIA1 promote tau phase separation *in vitro* without artificial crowding agents such as polyethylene glycol (PEG), supporting the potential physiological relevance of this process (Ash et al., 2021). PTMs provide another regulatory layer. Phosphorylation alters the charge distribution of tau and modulates its phase separation behavior by shifting interactions from electrostatically-driven to hydrophobically-driven mechanisms. Hyperphosphorylated tau condensates are therefore less dependent on salt and more sensitive to hydrophobic disruption (Bhandari et al., 2026).

In PD models, α-syn LLPS drives fibril formation through condensate maturation and the conversion of liquid-to-solid condensates. The α-syn NAC region is critical to both condensation and amyloid assembly and is believed to contribute to the transition from LLPS to LSPS (Han et al., 2024; Wasim et al., 2024; Wei et al., 2025). Synapsin condensates, which organize synaptic vesicle clusters at presynaptic terminals, partition α-syn under physiological conditions while maintaining its dynamic behavior. Their formation is regulated by the concentration, valency, and affinity of synapsin–α-syn interactions. Disruption of this balance may alter α-syn structural organization and physical phase, thereby contributing to pathological aggregation (Yasuda et al., 2013; Sharma and Burré, 2023; Ruiz-Ortega et al., 2024). Moreover, *in vitro* studies by Sawner et al. (2021) showed that ⍺-syn phase separation and aggregation can diverge at neutral pH and different salt concentrations, showing that this process is strongly influenced by the local environment (Sawner et al., 2021). Furthermore, Ray et al. (2020) demonstrated that α-syn LLPS precedes amyloid aggregation, with liquid-like condensates undergoing maturation into solid amyloid assemblies containing oligomers and fibrils. Disease-associated factors, including low pH, pathogenic mutations, and phosphomimetic modifications, enhance LLPS and promote its transition toward pathological aggregates both *in vitro* and in cells (Ray et al., 2020). While α-syn-LLPS and aggregate formation have been difficult to study independently because condensates rapidly transition into solid amyloid fibrils Mahapatra and Newberry (2024) uncoupled these events by decreasing α-syn sequence complexity, suggesting that LLPS and fibrillation can be independently regulated (Mahapatra and Newberry, 2024).

The transition from physiological LLPS to pathological aggregation can lead to loss of endogenous protein function, toxic gain of function through fibril formation, and propagation of pathology via self-seeding mechanisms (Xu et al., 2010; Vasili et al., 2019; Dhavale et al., 2024). Yet condensate properties do not map to pathology through any single means. Condensate formation and neuronal toxicity can be uncoupled, suggesting that LLPS may be permissive rather than a driving factor in disease (Fischer et al., 2026). Thus, the disease relevance of LLPS is determined not by condensate formation alone, but by the sequence- and context-dependent transitions that generate toxic, seed-competent assemblies.

## Discussion

Biomolecular condensates provide a framework for understanding how tau and α-syn transition from functional assemblies to pathological aggregates. Their material properties are governed by molecular interactions that support cellular function but can be altered by disease-associated changes, promoting aberrant protein assembly and neurodegeneration (Kanaan et al., 2020; Rai et al., 2021; Hou et al., 2023; Nam and Gwon, 2023).

Protein structure governs both the propensity of tau and α-syn to undergo LLPS and whether condensates remain reversible or mature into pathogenic assemblies, with electrostatic interactions in the tau N-terminal region driving phase separation and hydrophobic interactions within the MTBD contributing to condensate stabilization. The MTBD also contains PHF6 motifs that promote β-sheet formation and serve as nucleation sites for the transition to pathological tau aggregates (Wegmann, 2019; Rai et al., 2021). In α-syn, the NTD, NAC region, and acidic CTD regulate membrane interactions, self-association, and condensate behavior (Hardenberg et al., n.d; Xu et al., 2025; Arora and Wittung-Stafshede, 2026). Along with intrinsic sequence properties, cellular changes and PTMs can reshape intra- and intermolecular interactions, promoting the transition of tau and α-syn condensates from dynamic liquid states toward persistent, aggregation-prone assemblies that contribute to pathological fibril formation (Ray et al., 2020; Xu et al., 2023; Chen et al., 2025).

Although LLPS can serve physiological functions, disease-associated alterations in tau and α-syn can disrupt condensate homeostasis, promoting the emergence of less dynamic assemblies that facilitate pathological aggregation (Alavi Naini and Soussi-Yanicostas, 2015; Jonchhe et al., 2022; Foressi et al., 2023; Dogra and Mukhopadhyay, 2024). Nevertheless, the role of LLPS in neurodegeneration remains unclear, as condensate formation alone does not necessarily determine pathological outcomes (Alavi Naini and Soussi-Yanicostas, 2015; Jonchhe et al., 2022; Foressi et al., 2023; Dogra and Mukhopadhyay, 2024). Moreover, interpretation of LLPS studies requires caution, as many *in vitro* systems rely on molecular crowders (Shtilerman et al., 2002; Haduła et al., 2025) and simplified conditions that do not fully capture the complexity of neuronal environments, while direct measurement of condensate phase properties in living cells remains technically challenging. Unlike simplified *in vitro* systems, cellular phase behavior is continuously influenced by nucleic acids, PTMs, mutations, and active transport and turnover mechanisms. These processes collectively govern LLPS dynamics and regulate maturation by reshaping molecular interactions that dictate condensate stability, maturation, and conversion into less dynamic assemblies (Navalkar et al., 2025). Whether these alterations initiate pathogenic processes or represent downstream responses to neuronal stress and degeneration remains unresolved (Elbaum-Garfinkle, 2019). Furthermore, quantitative measurements of condensate material states in living cells remain constrained by indirect readouts and methods that can perturb normal cellular processes; for example, reagents such as 1,6-hexanediol or urea, commonly used to disrupt recombinant condensates (Poudyal et al., 2023), can produce secondary cellular effects that complicate in-cell interpretations. Despite extensive research efforts, AD and PD continue to pose significant clinical challenges, as the molecular drivers of disease progression and effective disease-modifying therapeutic strategies remain largely elusive (Nguyen and Derreumaux, 2026). LLPS illuminates transitions from functional protein organization to disease-associated states that underlie neurodegeneration (Chakraborty and Zweckstetter, 2023).
